# Adaptation of a Protocol to Evaluate Online Recruitment Strategies for Mailing HIV Self-Tests and Pilot an Intervention to Improve Linkage to HIV Prevention and Care Among Transgender Women: Protocol for a Randomized Controlled Trial

**DOI:** 10.2196/75345

**Published:** 2026-02-20

**Authors:** Joanna A Caldwell, Ruth Dana, Irah L Lucas, Patrick Sean Sullivan, Evelyn Olansky, Stephen Sullivan, Marissa Hannah, Travis Sanchez, Lisa Hightow-Weidman, Akshay Sharma

**Affiliations:** 1 Rollins School of Public Health, Department of Epidemiology, Emory University, Address 1518 Clifton Rd NE, Atlanta, GA, United States, +1 404 727 6123; 2 a Subsidiary of DLH Corp, Social and Scientific Systems, Inc, Atlanta, GA, United States; 3 Medical School, University of Michigan – Ann Arbor, 1109 Geddes Ave, Ann Arbor, MI, United States; 4 Department of Health Behavior and Clinical Sciences, University of Michigan, School of Nursing, , 1109 Geddes AveAnn Arbor, MI, United States

**Keywords:** HIV, HIV prevention, transgender women, linkage to care, HIV self-testing, protocol

## Abstract

**Background:**

Transgender women are disproportionately affected by HIV in the United States. Evidence shows that HIV self-testing increases awareness of HIV status, preventing transmission. Culturally appropriate strategies are needed to provide HIV self-tests (HIVSTs) to transgender women.

**Objective:**

This paper aims to describe an adaptation for transgender women of a comparative effectiveness trial of an intervention (Know@Home website) on the use of HIVSTs, counseling, and linkage of participants to appropriate services initially designed to reach Black or African American and Hispanic or Latino men who have sex with men (Implementation of Rapid HIV Self-Testing Among MSM Project).

**Methods:**

The ADAPT-ITT (Assessment, Decision, Adaptation, Production, Topical Experts, Integration, Training, and Testing) framework guided the adaptation. Project staff worked with consultants and a community advisory board to revise all study materials (ie, intervention web content, marketing text and images, and language used in the surveys) to be tailored to, and inclusive of, health topics relevant to transgender women. We conducted a pilot study with transgender women recruited through online venues. Participants were randomly assigned 2:1 to the intervention arm or control arm. All participants were mailed 2 HIVSTs, the OraQuick In-Home HIV Test, and had access to a standard resource list including information on how to find a nearby testing location, HIV prevention, and sexual health. Only intervention arm participants had access to the study website, “Know@Home.org.” The Know@Home website allowed participants in the intervention arm to order sexually transmitted infection test kits, condoms, and lubricant and provided HIV prevention information, service locators, and risk assessment tools. Adaptations to the intervention included modifying language, images, and links to outside resources to be transgender specific or gender neutral. Online surveys were adapted by removing inappropriate gender terms and replacing them with culturally appropriate terms for gender identity, genitalia, and condom use. Four months after completing the baseline (enrollment) survey, all participants were asked to complete a final follow-up survey. Upon completing the 4-month survey, participants were mailed a dried blood spot collection kit to be returned by mail for the laboratory testing of the sample. The results of all HIVSTs could be reported in real time in an online survey. Video health counseling was available, upon request, for all participants during study participation.

**Results:**

Enrollment into the pilot study began on April 22, 2021, and concluded on July 7, 2021, yielding a total of 102 transgender women participants, with data collection completed in January 2022.

**Conclusions:**

By using the ADAPT-ITT model, we produced materials to be culturally and linguistically appropriate for transgender women. The findings of this study have the potential to inform future research studies among transgender women and underscore the importance of involving subject matter experts and community members in the development of tailored materials.

## Introduction

The Ending the HIV Epidemic in the United States initiative is built on 4 science-based strategies. The first is diagnosing all individuals with HIV as early as possible. HIV testing allows people to learn their HIV status and initiate pre-exposure prophylaxis (PrEP) or antiretroviral treatment, preventing new infections. HIV self-testing (HIVST) is one component of this testing strategy [1-3].

In 2019, approximately 2% of HIV infection diagnoses were among transgender persons. The majority (93%) of these were among transgender women [4]. Transgender women in the United States are disproportionately affected by HIV with a prevalence of 14.1%, compared with an estimate of less than 0.5% for all US adults [5-7].

The underutilization of HIV testing may contribute to the disproportionate impact of HIV among transgender women [7], as HIV testing is the entry to engagement with HIV prevention and treatment [8]. A study in the United Kingdom found that transgender people are less likely to have ever tested for HIV than cisgender people (49% vs 64%, respectively) [9]. Barriers to HIV testing reported by transgender women include low risk perception, lack of access to HIV testing, and concerns of HIV-related stigma and transphobia in health care settings [10-12]. Providing HIVSTs to transgender women may reduce some barriers they experience when seeking gender-affirming HIV testing services by expanding routine testing options and partially mitigating the barrier of medical mistrust [10,13-15]. Self-testing allows users the flexibility of not having to present to clinical or community-based testing sites where providers may not be culturally competent. This testing approach provides an alternative to those who are distrustful of medical settings, who want to avoid settings that could trigger gender dysphoria, or who do not wish to be seen attending testing sites, due to stigma, discrimination, homophobia, transphobia, and racism [16,17].

Many studies conflate cisgender men who have sex with men (MSM) with transgender women, and the studies that disaggregate data on transgender women are limited [18,19]. Tailoring existing interventions is an important strategy to expedite the creation and deployment of effective interventions and improve public health outcomes for targeted populations [18,20,21]. Preliminary evidence indicates that technology-based behavioral health interventions, such as using social media, have the potential to promote HIV testing and decrease prevention disparities [22]. Social media–based interventions can increase engagement in care [23], and computerized counseling and text-based interventions can improve viral suppression among transgender women [24-27], although some studies did not show statistically significant results, and more research is needed [28].

This paper presents the adaptation of the study protocol and materials from the Implementation of Rapid HIV Self-Testing Among MSM Project (iSTAMP) [29] for use with transgender women. The iSTAMP study evaluated strategies to reach participants for distributing HIVST and incorporated a pilot evaluation of an HIV prevention phone app to improve linkage to HIV prevention and care designed for Black and Hispanic MSM [30]. Mailing HIVSTs to gay, bisexual, and other MSM increased rates of testing and awareness of infection status. An adaptation of the original protocol for transgender women was necessary to consider social and behavioral characteristics unique to this population.

Through an existing cooperative agreement with the Centers for Disease Control and Prevention, we obtained funds for conducting pilot activities for transgender women. This pilot project was funded separately from the original iSTAMP study at the end of year 3 of the 4-year project period.

The study aimed to evaluate (1) the comparative effectiveness of the intervention (Know@Home website) on the use of HIVSTs, counseling, and linkage of participants to appropriate services (ie, HIV treatment, HIV PrEP, sexually transmitted infection [STI] testing, additional prevention, and social services) and (2) the cost of implementing recruitment strategies and the intervention. We describe a community-engaged process to adapt the iSTAMP protocol to be culturally appropriate for transgender women, which included adapting recruitment materials, survey tools, and the interactive intervention website.

## Methods

### Overview

This pilot study assessed whether the iSTAMP protocol, online advertisements, and recruitment materials [30], originally designed for recruiting and engaging Black and Hispanic MSM, could be adapted for and implemented for transgender women. Most study procedures were the same as those described in the iSTAMP protocol paper [30]. Briefly, the iSTAMP randomized control trials (both the original study and this adaptation) were designed to evaluate a program that distributed HIVST kits. The iSTAMP included a nested randomized component assessing the utility of comprehensive prevention content (Know@Home [31]) to support related HIV prevention services for those assigned only to the intervention arm. The adaptation substituted the Know@Home mobile app for a website named Know@Home with similar materials tailored to transgender women. The intervention content included HIV prevention information; a way to order condoms and STI self-collection kits; risk assessment tools; and locators for HIV testing, HIV treatment, and PrEP services. Four months after completing the baseline (enrollment) survey, all participants were asked to complete a final follow-up survey, which collected information on sexual behavior, HIV testing and status, and linkage to care. Upon completing the 4-month survey, participants were mailed a dried blood spot collection kit to be returned by mail for laboratory testing. The results of all HIVSTs could be reported in real time into an online survey. Video health counseling was available, upon request, for all participants during study participation. Further details about study design, participant flow, data collection, and planned analyses can be found in the original iSTAMP protocol paper [30].

This paper focuses on materials affecting the transgender participant, rather than internal staff documents, such as standard operating procedures and email, text, or phone scripts. Electronic case report forms captured enrollment and retention, HIVST result follow-up communications, referrals, and video counseling.

Our adaptation was guided by the 8 phases of the ADAPT-ITT (Assessment, Decision, Adaptation, Production, Topical Experts, Integration, Training, and Testing) model [32] (Table 1). We chose the iSTAMP study for assessment, reviewed literature, identified domains of the study for adaptation, and developed a list of needed adaptations. Topical experts provided input on marketing and recruitment materials and recommended changes to intervention materials and survey assessments. Suggested changes were integrated into all study materials. The testing phase of the pilot study and associated data analyses occurred as the final phase.

**Table 1.** Use of ADAPT-ITT (Assessment, Decision, Adaptation, Production, Topical Experts, Integration, Training, and Testing) framework for the development of a protocol for distribution of HIV self-test kits for transgender women in the United States (2020).

Phase	Staff roles	Activity	Outcomes
Assessment	Researchers	Literature review	Determined need for increased HIV testing options
Decision	Researchers	Literature review	Selected the iSTAMP^a^ protocol
Adaptation	Community consultants	Review of iSTAMP materials	Identified questions, images, and content for revision
Production	Researchers	Review of input from community consultants	Created required changes in recruitment materials, images, intervention materials, and survey assessments
Topical experts	Subject matter experts Community advisory board	Recommend revisions Formative discussion to provide feedback	Provided feedback on intervention materials and survey assessment Provided feedback on marketing, images, and recruitment materials
Integration	Research staff	Create new website and modify study assessments	Revised marketing materials, recruitment materials, intervention materials, and assessment
Training or testing	Researchers, research staff	Implement pilot study	Analyzed preliminary data on feasibility, acceptability, recruitment, retention, and HIV testing outcomes

a iSTAMP: Implementation of Rapid HIV Self-Testing Among MSM Project.

### Assessment

In the assessment phase, we reviewed relevant literature and decided to adapt an intervention to promote HIV self-testing to increase HIV testing and awareness of HIV status for transgender women. An informal literature review indicated that transgender women were disproportionately affected by HIV [6,7,33]. Transgender women’s unique experiences and their desire for HIV interventions and services necessitated specialized research to assess risks that differentiate transgender women from MSM [33,34].

The literature review indicated that additional behavioral health survey items, which are not typically included in HIV research with MSM, for example, gender identity and hormone replacement therapy, would be relevant for transgender women. These items were chosen and included in the survey tool. Research staff determined their capacity to adapt and implement the HIV counseling and referral component of the study and assessed the availability of transgender-specific referral resources for HIV testing and prevention services.

### Decision and Adaptation

In the decision phase, we selected the iSTAMP study because the distribution of HIV self-test kits has shown to increase HIV testing in MSM [35], and there were specifically designed materials and processes available that could be adapted.

In the adaptation phase, although many aspects of the original iSTAMP study protocol remained the same (eg, OraQuick In-Home HIV test, the recruitment, and mailing systems), several domains required revision, including the recruitment materials (images and text), survey questions, and an intervention website. In March 2020, 5 members of the community and several culturally competent iSTAMP study staff identified language in existing survey materials that was not culturally appropriate for transgender women. They provided alternative appropriate language. A US $50 gift card was provided for the community members’ time.

### Production

During the production phase, staff reviewed the relevant literature from the assessment phase and created a list of required changes. This was a preliminary list of potential adaptations required to tailor the iSTAMP study for transgender women.

### Topical Experts: Recruitment and Advertising Materials

Study staff convened an online meeting of consultants to identify effective recruitment strategies and tailor advertising materials specifically for transgender women. Consultants were either transgender women, transfeminine-identifying individuals, or nonbinary-identifying individuals. We recruited the consultants through social media and flyers sent to lesbian, gay, bisexual, transgender, and queer organizations located in the 11 states from which we planned to recruit participants. Each consultant was provided a US $50 e-gift card for their time. Four consultants participated in a recorded, online videoconference held in November 2020.

The consultants discussed the suitability of recruiting transgender women from online dating websites and social media platforms and explored potential platforms that may have had a greater transgender women user base than those already under consideration, such as TikTok. The facilitator presented the examples of advertising images and text messages to assess cultural acceptability.

Consultants identified that the pop cultural references used in the original intervention were more relevant to MSM and the drag community than to transgender women. The examples included language such as “Demon time” that references horniness and “Faster than beating your mug” that references applying makeup. The use of drag references with transgender women was deemed by the consultants to be offensive and inappropriate.

The language that alluded to using sex-seeking apps (eg, “You host, we’re mobile” appearing to reference the dating app Grindr, or “Plan for your fans” appearing to reference the website OnlyFans, which is often used by sex workers) was viewed negatively by consultants. The consultants stated that the transgender women’s community has often been portrayed as hypersexualized within mainstream media and that these text messages and images reinforced the stereotype. Advertisement text that focused on respecting transgender identities and intimated themes of empowerment was preferred.

Consultants emphasized the importance of using photographs with transgender women. Images where the individual’s transgender or gender nonconforming identity was more prominent were rated higher. Consultants stated that text specifically used to provide assurances of data security or confidentiality (eg, “Your identity is safe here”) increased the skepticism of data protections and how participant information may be used. Another identity-related concern the consultants discussed was that the study should consider developing procedures to allow participants to use different names in different contexts. For example, some participants may prefer to use their legal name or “deadname” at home because they are not “out.” They reported that participants might want to use a chosen name on email and text communications but use their legal name or “deadname” on postal packages to avoid disclosing their transgender identity to the people with whom they live.

**Figure 1.**
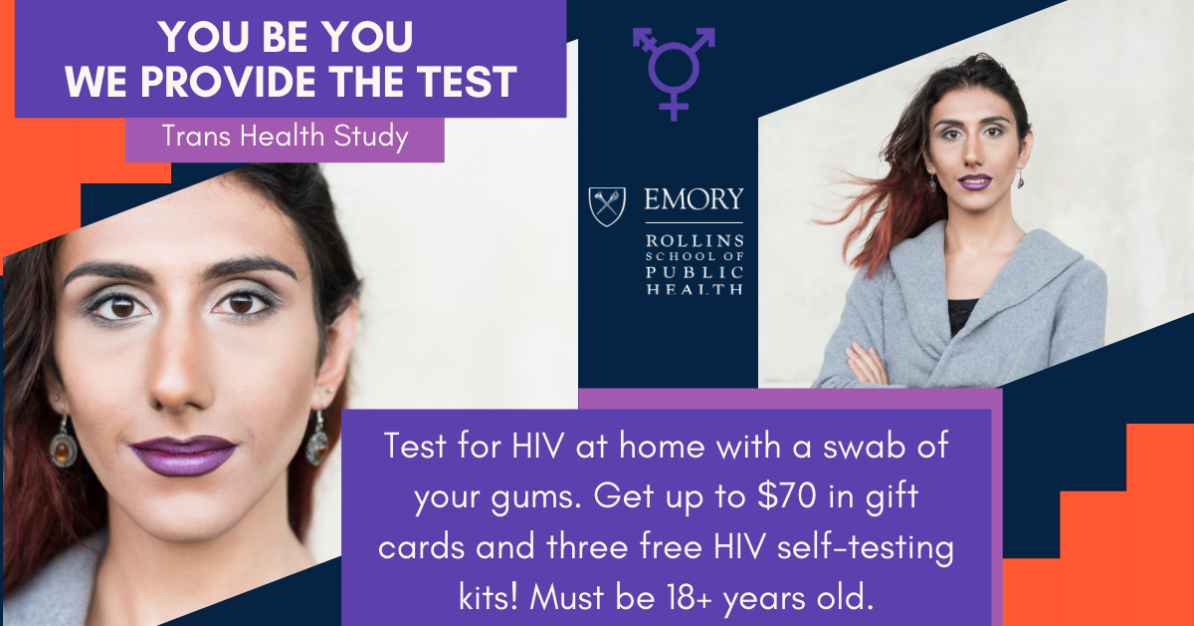
High-performing ad copy (sample 1).

There was a consensus among consultants that obtaining free HIVSTs and STI home collection kits would be viewed as beneficial by transgender women. Consultants also agreed that the financial token of appreciation for the participant’s time should be included in the advertisement because it may be viewed by many potential participants as immediately beneficial. Figures1  2 provide examples of recruitment advertisements that resulted in greater response from potential participants during the study. All advertisements were created using licensed stock photos.

**Figure 2.**
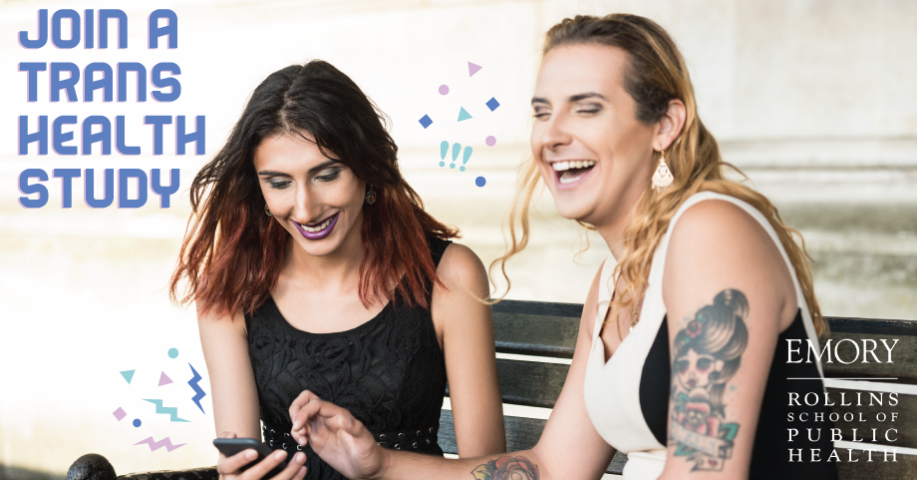
High-performing ad copy (sample 2).

### Topical Experts: Intervention Website

In this pilot study, we substituted the Know@Home mobile app, provided for the MSM intervention arm of the original iSTAMP trial, for a website named Know@Home, which included the same materials adapted for transgender women. The website contained HIV prevention information and allowed participants to order free materials (eg, condoms and STI self-collection kits) and access online risk assessment tools and service locators for HIV testing, HIV treatment, and PrEP services.

A subject matter expert (SME) on transgender health reviewed the Know@Home website intervention content and supporting materials. The expert proposed revisions to make the content suitable for transgender women based on recommendations from the Provincial Health Services Authority of British Columbia [36], which was a reliable source for recommendations at the time of study design. The reviewed intervention sections included written and video messaging components, risk-assessment quizzes to help assess one’s risk for HIV infection, embedded links to external pages, and the static informational pages consisting of the “About Us” page and STI and HIV information.

Materials were revised with the gender-neutral terms “person” and “people.” Materials that directly referred to sexual anatomy were revised to use gender-neutral anatomical terms [36]. Video messages and external links to study materials were reviewed, and those appropriate for MSM were replaced with gender-neutral alternatives. Content that featured only MSM was replaced with content that featured transgender women or images that would be appropriate for all audiences regardless of gender identity. Table 2 lists the 4 core themes identified as requiring revisions and examples of appropriate terminology replacements. The four core themes included: (1) gendered terminology referring to the participant, (2) gendered terminology referring to the participant’s sex partners, (3) terminology describing genitalia and sexual acts, and (4) condom use.

**Table 2.** Thematic content from the Implementation of Rapid HIV Self-Testing Among MSM Project (iSTAMP) study identified as not suitable for HIV self-test distribution for transgender women (2020).

Theme identified	iSTAMP terms and phrases	Revised terms and phrases	Reasons text modified	Study materials updated
Participant gender identity	Men, man, MSM^a^, guys Gay and bisexual men	People, person Transfeminine people, transgender women	Avoids misgendering transgender women	Website Surveys Advertisements Videos Images
Sex partner or partners gender identity	Men, man, MSM, guys	Partner, partners, sex partner(s)	Avoids potentially misgendering a participant’s sex partner or partners Avoids assumption that a participant has sex with only cisgender men	Website Surveys
Terms describing genitalia and sex acts	Penis, scrotum, testicles Penis, vulva Vagina Vaginal fluids, semen	Outer parts External pelvic area Internal pelvic area Genital fluids	Avoids biological terms associated with a specific gender that may cause discomfort or gender dysphoria for a participant or their partner or partners Avoids biological terms that may not be the preferred term of the participant or participant’s partner or partners	Website Surveys

a MSM: men who have sex with men.

### Topical Experts: Survey Assessments

An SME on transgender health reviewed the online surveys and identified items that needed to be rephrased with culturally appropriate terminology based on current best practices [37]. The SME identified and recommended survey domains that were relevant to transgender health that were not included in the original surveys designed for MSM.

Similar to the intervention materials, baseline and follow-up surveys were revised to ensure clarity and appropriateness of questions pertaining to the gender, sex, and genitalia of respondents and their sexual partner or partners. Questions were removed if they fell within the 4 core themes identified as inappropriate for transgender women. Supplemental questions related to the experiences of transgender women were developed using appropriate terminology for transgender women. These questions were provided to participants directly after they completed the baseline and follow-up surveys.

The supplemental questions obtained data on the following domains: gender identity, sexual orientation, gender presentation, mental health and suicidality, sexual behavior (using affirming language for genitalia), gender confirmation surgeries, hormone replacement therapy, electrolysis, hormonal and medical gender affirmation, gender-based stigma, and history of intimate partner violence. For transgender-specific intimate partner violence, we used the Peitzmeier Transgender-Specific Intimate Partner Violence scale, which is tailored for transgender women and consists of four domains: (1) coercive control of gender transition or gender presentation, (2) emphasizing the undesirability of transgender individuals as intimate partners, (3) blackmail via “outing,” and (4) sabotaging transition [38]. This tool has demonstrated adequate internal consistency (KR-20=0.56) [38]. To measure gender-based stigma, we used a 13-question scale developed by Maksut et al [39] that assessed perceived, anticipated, and enacted stigmas due to gender identity. This gender identity stigma scale has demonstrated acceptable internal consistency (Cronbach *𝑥*=0.79) [39].

### Integration and Testing

Project staff, the principal investigators, and SMEs reviewed and approved each adaptation. With the deployment of the redesigned website and the implementation of the revised materials for transgender women, we conducted a randomized control trial in which we aimed to recruit up to 200 participants. The eligibility criteria are described in Textbox 1.

Textbox 1. **Eligibility and exclusion criteria for an HIV self-test pilot study of transgender women living in 11 US states (2020-2021).**

**Eligibility criteria**

- Provided informed consent online
- At least 18 years of age
- Identifies as a transgender woman or transgender female
- Assigned male sex at birth
- Has an Android or iOS mobile smartphone with active service
- Resides in Alabama, California, Florida, Georgia, Louisiana, Mississippi, North Carolina, Nevada, New York, South Carolina, and Texas
- Provides valid contact information
- Successfully completed the baseline survey

**Exclusion criteria**

- Declined consent to participate
- Currently participating in another HIV prevention research study or program
- Has a bleeding disorder
- Participant in an HIV vaccine study
- Currently taking pre-exposure prophylaxis for HIV prevention
- Reported a prior positive HIV test result
- Plans to move out of a study state during the 4 months study period

The goal of the pilot study was to provide preliminary data on recruiting transgender women online and the feasibility of enrolling transgender women into an HIV self-testing study. The data collected provide estimates for the length of time needed to recruit participants, the effectiveness of online recruitment strategies, the cost of recruitment, retention rates, the use and acceptability of video counseling services, and the proportion of transgender women who reported using the HIVSTs and STI self-collection kits. These data will be used in conducting power calculations for future HIV self-testing research studies with transgender women.

We planned to use the data to identify key considerations for programmatic implementation of a mail-out HIV self-testing program for transgender women that could be implemented by local health departments or community-based service providers. Factors that are relevant for implementation by these providers include online recruitment and costs to implement.

For this pilot study, potential participants were recruited through online venues, such as social networking sites (eg, Facebook) and dating apps (eg, Jack’d), which were chosen using advertising data from the previous recruitment of transgender women. Participants completed an initial eligibility screening survey, provided electronic informed consent, and completed a baseline survey. An initial validation process was conducted to determine enrollment status. Participants were considered fully enrolled in the study and mailed 2 HIVSTs after they had provided the required contact information, and duplicate accounts were excluded.

We used a two-stage stratified randomization procedure [40]. First, we grouped participants based on race or ethnicity (Black, White, Other, Hispanic or Latina) for a total of 4 strata. Next, we randomly assigned participants from each strata to the intervention arm (mailed self-test kit+provided link to the Know@Home website) or control arm (mailed self-test kit only) with a 2:1 ratio.

### Ethical Considerations

This pilot study was approved by the Emory University Institutional Review Board (IRB00099710) and is registered at ClinicalTrials.gov (NCT04219878). Personal identifying information was only stored at Emory University. Confidential information was stored off site on secure web servers. All data files were encrypted with strong password protection. Access to personally identifying information was restricted to Emory study staff. Informed consent was acquired online. Incentives in the form of e-gift cards were provided to participants for completing various study activities totaling up to US $70 over the 4-month study period. Additional optional surveys allowed participants to earn an additional US $60.

## Results

The main research outcomes from this pilot study are summarized in Table 3. During the enrollment period, from April 22, 2021, to July 7, 2021, a total of 2014 people consented to be screened for the study, most of whom were ineligible for multiple reasons. Not identifying as female or a transgender woman was the most common reason for ineligibility. Of 217 people who were eligible based on screening criteria, 169 provided contact information. Of these, 108 participants passed the initial validation process, responded to a text message to complete the validation process, and completed the baseline survey. Subsequently, we determined that an additional 6 people had provided false information (eg, they revealed that they did not identify as transgender women) and were excluded from the analyses. A total of 102 transgender women constituted the sample for this pilot study, and 81 provided follow-up information. In total, 20 participants (18%) were non-Hispanic Black, 49 (48%) were non-Hispanic White, 18 (17%) were Hispanic or Latina of any race, and 15 (14%) were of another race.

**Table 3.** Research outcomes, data types, and relevance to study design implementation for HIV self-test kit distribution for transgender women, 11 US states, 2020.

Research outcomes	Types of data	Relevance to study design
Feedback on sample advertisements	Qualitative report from consultant’s video conference	Allows for more effective and culturally appropriate recruitment
Proposed recruitment strategies for transgender women (specific online sites, influencers, advertisement images, language)	Cost data Advertisement click-through rates Eligibility and enrollment counts based on venue and advertisement copy	Allows for cost analysis for recruitment venues
Feasibility of online enrollment of transgender women	Characteristics of transgender women enrolled Ability to obtain target enrollment	Allows for assessment of potential biases in recruitment of transgender women (eg, by age, region, race, or ethnicity) and development of plans to mitigate selection biases and allows ability to determine if sample size will be met
Time to recruit	Time required to recruit transgender women	Allows for planning of appropriate recruitment period and funds for recruitment
Retention rates	Stratified retention estimates by age Region Race or ethnicity	Allows for planning of sample size of a large-scale trial, accounting for retention
Percent of participants using study HIVST^a^	Reported use of HIVST kits Distribution of HIVST kits to others Reasons for using or not using kits	Allows for sample size calculations and budgeting and number of HIVST kits required for distribution
HIV prevalence among pilot study participants	Reported results of HIVST	Allows for estimation of initial sample size to develop a cohort of transgender women who have a negative HIV status for prospective research; allows planning for needs for video counseling resources
Percent of participants using video counseling services	Number of counseling sessions requested, scheduled, and attended Client preferences for time of day and day of week	Allows for planning of the number of counselors needed and meeting the capacity for counseling sessions each week based on preferences for time of day and day of week

a HIVST: HIV self-testing.

## Discussion

### Principal Findings

This paper documents the process of adapting the iSTAMP protocol developed for Black and Hispanic MSM to be appropriate for engaging transgender women, using the 8-phase ADAPT-ITT model. The purpose of this adaptation is to ensure that study procedures and materials presented to participants are culturally and linguistically appropriate and do not cause discomfort or harm. In our adaptation process, we strove to ensure that study materials would not misgender participants and that questions and responses would be culturally relevant and inclusive [41,42]. These adaptations were crucial to ensure the data obtained were relevant for transgender women who participated in the study and would allow us to gain a comprehensive understanding of the experiences of transgender women. Other adaptation studies reflected the importance of including content related to gender dysphoria, police or law enforcement interactions, HIV testing, PrEP, and antiretroviral treatment as well as reflections of transgender women in the study materials [43]. Several studies used focus groups or community advisory boards, including transgender members [43-45]. Some studies noted the importance of including transgender women at any stage of transition as well as nonbinary individuals [43,44]. The resources developed in this protocol can be used to provide preliminary data and tools for further development and evaluation of a robust trial HIVST distribution and linkage to prevention services for transgender women in the United States.

### Limitations

We encountered several challenges during the development and implementation of this pilot study. This activity was conducted after the completion of a trial for MSM with supplemental funding awarded in the final year of the original study. Consequently, the achieved sample size was half of what was originally planned because there were time constraints in which all activities had to be completed, leading to potential limitations around the ability to assess feasibility. Sample representativeness is limited by sampling technique and sample size.

Only 4 of the 9 consultants were able to participate in the online videoconference due to scheduling conflicts, limiting the input on the adaptation process; however, written feedback was elicited from all 9 consultants, and we had coauthors who are SMEs who contributed to the adaptation. The study was conducted in 11 US states, predominantly located in jurisdictions with a high prevalence of HIV in the United States. The sample does not represent all transgender women in the United States.

Due to time constraints, we created a web-based version of the original intervention developed for use on a mobile phone. We used the same eligibility screener questions from the MSM trial due to timeline constraints, and participants were required to identify as “male to female transgender.” This language may have discouraged some transgender women from participating in the study if they did not identify with this terminology. To earn trust among the community, it is necessary to use appropriate language [41], and future studies can incorporate an expanded eligibility criterion to include transgender women who may identify with alternative terminologies, such as transgender woman, transfeminine, or other terms that are commonly used at the time of study implementation.

This study examines a mobile intervention, which is accessible to people with smartphones. Additionally, nearly half of the study population is non-Hispanic White. Although over 91% of the US population owns a smartphone [46] and over half the US population is non-Hispanic White, these factors could have led to an underrepresentation of groups bearing a high HIV burden among transgender women.

### Future Adaptations

During the adaptation process, we identified several key principles that may be useful for future adaptations of protocols or interventions for transgender women. When collecting names and pronouns, researchers can inform participants that legal names may not be required to participate, and when possible, collect both the legal name and the name the participant wishes to use during study participation. Engaging members and representatives of the community in planning the research design and materials can mitigate cultural competency issues when tailoring materials. Involving SMEs on transgender women, particularly when the experts are a part of the intended study population, in planning the research design and materials for future studies for transgender women could allow studies to be culturally appropriate [47-50].

Mixed methods approaches provide an opportunity for increasing cultural competency of materials and survey instruments. Qualitative methods can be used to allow transgender women to provide detailed feedback on materials and terminology before implementation. Validating survey instruments that were designed for other populations as well as formal A or B testing would improve recruitment optimization and study rigor.

Linguistic choices that pathologize or marginalize people are potentially harmful to participants during the research and publication of findings [41,51]. As languages and cultures evolve, it is necessary to adapt terminology so that terms are culturally appropriate for the proposed populations. This may require modifying demographic terminology (Table 2) used to collect data to reflect the culture of the population. Finally, while this pilot study engaged transgender women, future studies could widen inclusion criteria to include other populations who may also benefit from HIV self-test programs, such as nonbinary people, gender-nonconforming people, transgender men, and transmasculine people.

### Conclusions

We adapted study materials for an HIV prevention intervention for transgender women. By using the ADAPT-ITT model, we systematically produced materials that are culturally and linguistically appropriate for transgender women. The research process and resulting materials have the potential to inform future research studies among transgender women and underscore the importance of involving SMEs and community members in the development of tailored materials.

## Abbreviations

ADAPT-ITT: Assessment, Decision, Adaptation, Production, Topical Experts, Integration, Training, and Testing
HIVST: HIV self-test
iSTAMP: Implementation of Rapid HIV Self-Testing Among MSM Project
MSM: men who have sex with men
PrEP: pre-exposure prophylaxis
SME: subject matter expert
STI: sexually transmitted infection
